# PKM2 promotes glucose metabolism and cell growth in gliomas through a mechanism involving a let-7a/c-Myc/hnRNPA1 feedback loop

**DOI:** 10.18632/oncotarget.3514

**Published:** 2015-02-10

**Authors:** Wenkang Luan, Yingyi Wang, Xincheng Chen, Yan Shi, Jiajia Wang, Junxia Zhang, Jin Qian, Ri Li, Tao Tao, Wenjin Wei, Qi Hu, Ning Liu, Yongping You

**Affiliations:** ^1^ Department of Neurosurgery, The First Affiliated Hospital of Nanjing Medical University, Nanjing, China; ^2^ Department of Neurosurgery, People's Hospital of Xuancheng City, Anhui, China

**Keywords:** let-7a microRNA, c-Myc, hnRNPA1, PKM2, glucose metabolism, aerobic glycolysis, glioma

## Abstract

Tumor cells metabolize more glucose to lactate in aerobic or hypoxic conditions than non-tumor cells. Pyruvate kinase isoenzyme type M2 (PKM2) is crucial for tumor cell aerobic glycolysis. We established a role for let-7a/c-Myc/hnRNPA1/PKM2 signaling in glioma cell glucose metabolism. PKM2 depletion via siRNA inhibits cell proliferation and aerobic glycolysis in glioma cells. C-Myc promotes up-regulation of hnRNPA1 expression, hnRNPA1 binding to PKM pre-mRNA, and the subsequent formation of PKM2. This pathway is downregulated by the microRNA let-7a, which functionally targets c-Myc, whereas hnRNPA1 blocks the biogenesis of let-7a to counteract its ability to downregulate the c-Myc/hnRNPA1/PKM2 signaling pathway. The down-regulation of c-Myc/hnRNPA1/PKM2 by let-7a is verified using a glioma xenograft model. These results suggest that let-7a, c-Myc and hnRNPA1 from a feedback loop, thereby regulating PKM2 expression to modulate glucose metabolism of glioma cells. These findings elucidate a new pathway mediating aerobic glycolysis in gliomas and provide an attractive potential target for therapeutic intervention.

## INTRODUCTION

A change in energy metabolism is listed as one of the ten hallmarks of cancer.[1] Under aerobic conditions, normal differentiated cells extract energy from glucose chiefly through oxidative phosphorylation, but tumor cells metabolize more glucose to lactate. This phenomenon, termed the Warburg effect (aerobic glycolysis), is important for tumor cell proliferation.[2, 3] Glioma, the most common primary brain tumor, also exhibits the Warburg effect.[4-6] However, few studies on glioma cell metabolism have been reported.

Pyruvate kinase is the most important rate-limiting enzyme of glycolysis. Pyruvate kinase isoenzyme type M1 (PKM1) and M2 (PKM2) are two isoforms of pyruvate kinase. The PKM1 promotes oxidative phosphorylation, whereas PKM2 primarily contributes to aerobic glycolysis.[7-9] The expression of PKM2 is increased in many tumors, including gliomas.[10-12] PKM1 and PKM2 are produced by alternative splicing of transcripts of the PKM gene. PKM1 and PKM2 differ in the inclusion of exon 9 or 10. PKM1 exclusively contains exon 9 whereas PKM2 exclusively contains exon 10. During splicing, three heterogeneous nuclear ribonucleoprotein (hnRNP) proteins-- hnRNPA1, hnRNPA2 and polypyrimidine tract binding protein (hnRNPI, PTB)--bind repressively to exon 9 to facilitate the exclusion of exon 9 and the generation of PKM2 in glioma.[12]

Our previous data have shown that let-7a microRNA suppresses its target transcript K-ras and inhibits glioma malignancy independent of PTEN.[13] In the current study, we investigated the role of let-7a and PKM2 in the glucose metabolism of glioma cells. We demonstrated that let-7a, c-Myc and hnRNPA1 form a feedback loop to modulate PKM2 expression, thereby influencing glioma cell glucose metabolism and growth. Thus, the let-7a/c-Myc/hnRNPA1/PKM2 signaling pathway may serve as the potential target for glioma therapy.

## RESULTS

### PKM2 promotes aerobic glycolysis and proliferation of glioma cells

**Figure 1:**
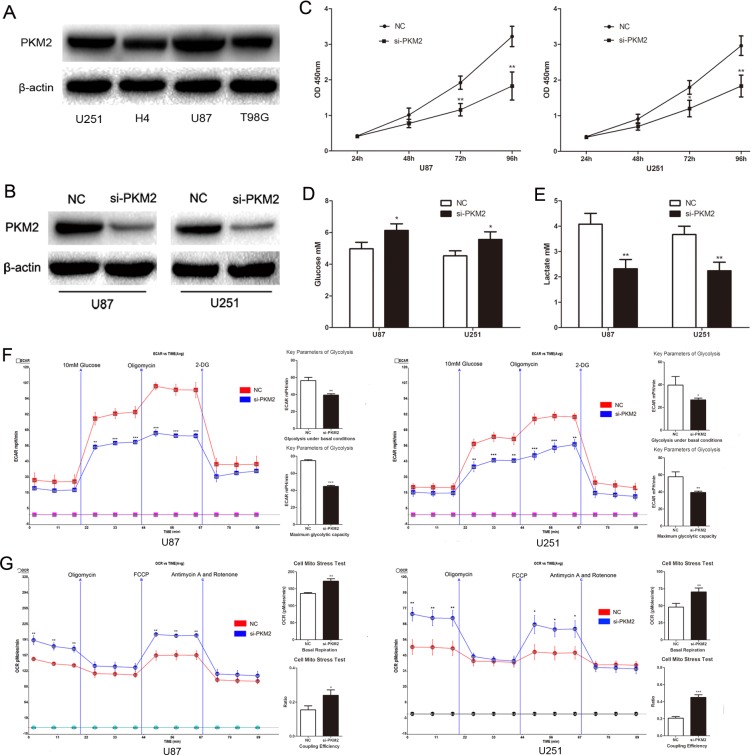
PKM2 promotes glioma cells aerobic glycolysis and proliferation (A) PKM2 expression profile in glioma cell lines, including U87, U251, H4 and T98G glioma cell lines. (B) Transfection efficiency of si-PKM2 was determined by Western blotting. (C) The proliferation ability of glioma cell lines U87 and U251 was measured by CCK8 assay. (D and E) The concentration of glucose and lactate in the culture medium was measured after the glioma cells were transfected with si-PKM2 or a negative control siRNA (NC). (F and G) ECAR and OCR were measured by the Glycolysis Stress and Cell Mito Stress tests in glioma cell lines after the cells were transfected with si-PKM2 or NC. *P < 0.05, **P < 0.01, ***P<0.001. Results are representative of at least three independent experiments.

To explore the role of PKM2 in glioma cells, we first detected PKM2 expression in glioma cell lines and found PKM2 was higher in U87 and U251 cells than in H4 and T98G cells. (Fig. 1A). In both U87 and U251 cells, decreased PKM2 significantly inhibited the proliferation ability of glioma cells (Fig. 1B and 1C). Decreased PKM2 also significantly inhibited glucose consumption, leading to elevated glucose levels and reduced lactate levels (Fig. 1D and Fig. 1E). Consistent with a role for PKM2 in extracellular acidification, si-PKM2 transfected cells exhibited lower levels of the extracellular acidification rate (ECAR) after treatment with glucose or oligomycin compared with the negative control: measurements of glycolysis under basal conditions and the maximum glycolytic capacity were both inhibited when PKM2 was decreased (Fig. 1F). Furthermore, PKM2 depletion resulted in increased levels of the oxygen consumption rate (OCR), an indicator of mitochondrial respiration, both under basal and carbonyl cyanide-p-trifluoromethoxyphenylhydrazone (FCCP) treated conditions (Fig. 1G). The coupling efficiency was higher in the si-PKM2 transfected cells (Fig. 1G). In addition, PKM2 overexpression in H4 and T98G cells promoted glucose consumption by reducing glucose levels and elevating lactate levels (Supplementary Fig.1). These findings support a role for PKM2 in promoting aerobic glycolysis and proliferation of glioma cells.

### Let-7a represses glioma cell glucose metabolism by inhibiting PKM2

**Figure 2:**
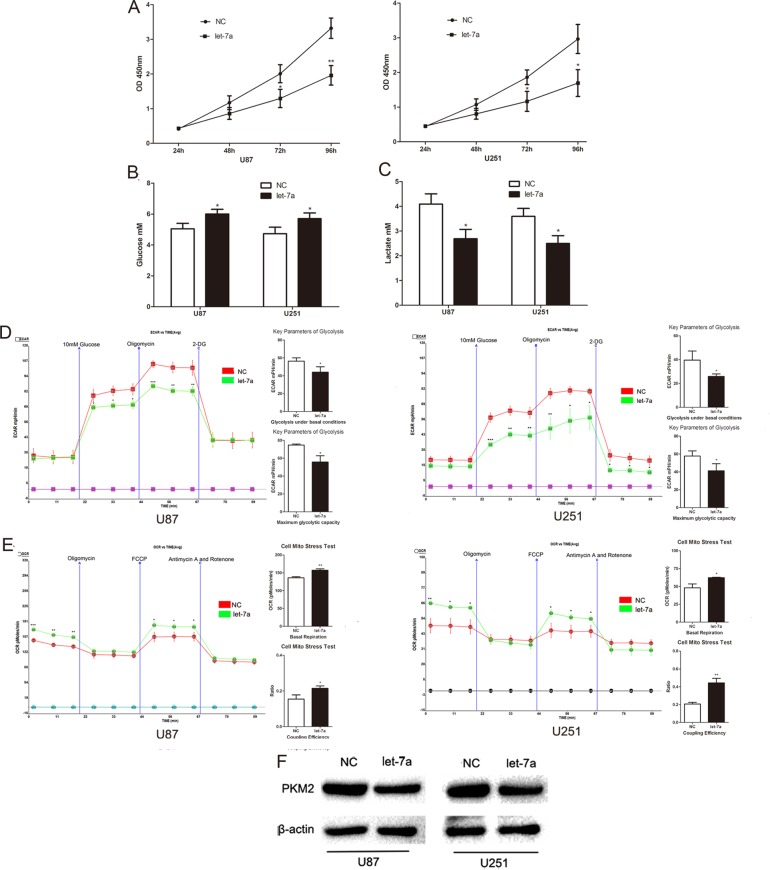
Let-7a represses glioma cell glucose metabolism by inhibiting PKM2 (A) The effect of let-7a on the proliferation ability of U87 and U251 glioma cell lines was measured by CCK8 assay. (B and C) The concentration of glucose and lactate in the culture medium was measured after the glioma cells were transfected with let-7a or a negative control miRNA (NC). (D and E) ECAR and OCR were measured by the Glycolysis Stress and Cell Mito Stress tests in glioma cell lines after the cells were transfected with let-7a or NC. (F) Overexpression of let-7a decreased the expression of PKM2 in U87 and U251 cells as assessed by Western blotting. β-actin is shown as a loading control. *P < 0.05, **P < 0.01, ***P<0.001. Results are representative of at least three independent experiments.

Recently, we demonstrated that let-7a induces cell apoptosis and inhibits cell proliferation, migration and invasion in gliomas.[13] Based on the well-characterized relationship between glucose metabolism and proliferation,[2, 3] we postulated that let-7a might inhibit glioma cell proliferation through a mechanism involving glucose metabolism. Consistent with previous observations, expression of let-7a mimic inhibited the proliferation of U87 and U251 glioma cells (Fig. 2A). Furthermore, the concentration of glucose in culture medium was increased and the concentration of lactate was decreased in let-7a mimic-transfected cells (Fig. 2B and Fig. 2C), suggesting that the reduction in proliferation may be related to the inhibition of glucose metabolism. Consistently, ECAR and OCR data showed that the rate of glycolysis under basal conditions and maximum glycolytic capacity were decreased, whereas the basal respiration and the coupling efficiency were increased in let-7a-transfected cells (Fig. 2D and Fig. 2E). Importantly, increased let-7a repressed PKM2 expression in glioma cells (Fig. 2F). These data indicate that let-7a may affect aerobic glycolysis through PKM2 in glioma cell lines.

### HnRNPA1 is critical for the generation of PKM2 in glioma cells

**Figure 3:**
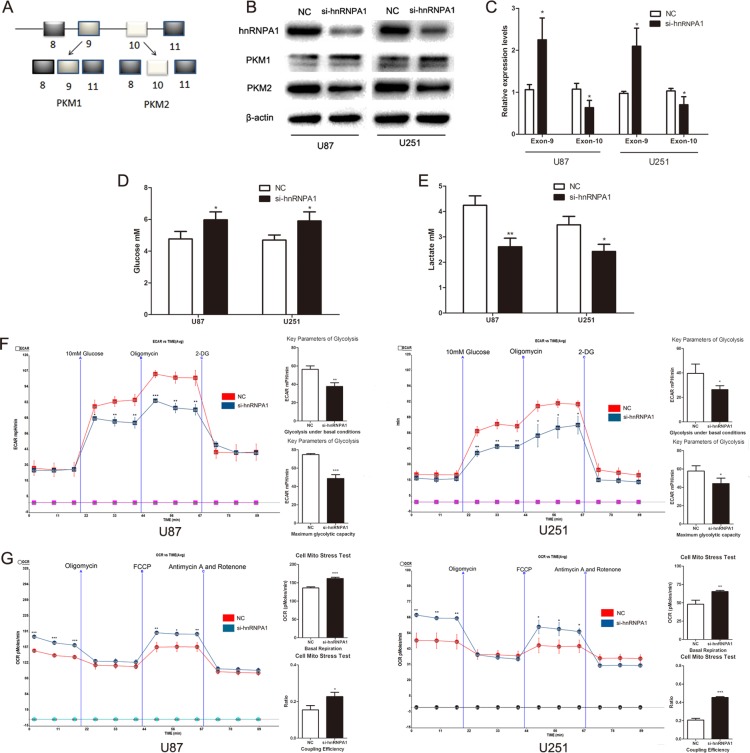
HnRNPA1 is critical for the generation of PKM2 in glioma cells (A) Schematic diagram of PKM splicing. hnRNPA1 promotes the formation of PKM2 (which includes exon 10) rather than the formation of PKM1 (which includes exon 9). [12] (B) Western blots identified hnRNPA1, PKM1 and PKM2 expression changes following transfection of U87 and U251 glioma cells with si-hnRNPA1. β-actin is shown as a loading control. (C) The expression of exon 9 and exon 10 were determined by qRT-PCR following transfection of si-hnRNPA1 or negative control siRNA (NC) into U87 and U251 cells. The expression of exon 9 and exon 10 was normalized to the expression of GAPDH mRNA. (D and E) The concentration of glucose and lactate in the culture medium was measured after the glioma cells were transfected with si-hnRNPA1 or NC. (F and G) ECAR and OCR were measured by the Glycolysis Stress and Cell Mito Stress tests in glioma cell lines after the cells were transfected with si-hnRNPA1 or NC. *P < 0.05, **P < 0.01, ***P<0.001. Results are representative of at least three independent experiments.

HnRNPA1 binds repressively to exon 9 to promote the generation of PKM2 (Fig. 3A).[12] To verify a role for hnRNPA1 in PKM2-mediated regulation of glucose metabolism we used siRNA to down-regulate hnRNPA1 expression in glioma cells. As expected, the expression of si- hnRNPA1 led to a decrease in PKM2 protein expression and an increase in PKM1 protein expression (Fig. 3B). To verify these results, we designed specific primers for exon 9 and exon 10. qRT-PCR confirmed that the exon 9 was up-regulated and exon 10 was down-regulated in si- hnRNPA1-transfected cells (Fig. 3C). Furthermore, si-hnRNPA1 significantly inhibited glucose consumption and lactate generation (Fig. 3D and Fig. 3E), and led to lower ECAR and higher OCR levels (Fig. 3F and Fig. 3G). These data indicate that hnRNPA1 is critical for the generation of PKM2, which regulates glucose metabolism in glioma cells.

### C-Myc regulates PKM2 by directing the transcription of hnRNPA1

**Figure 4:**
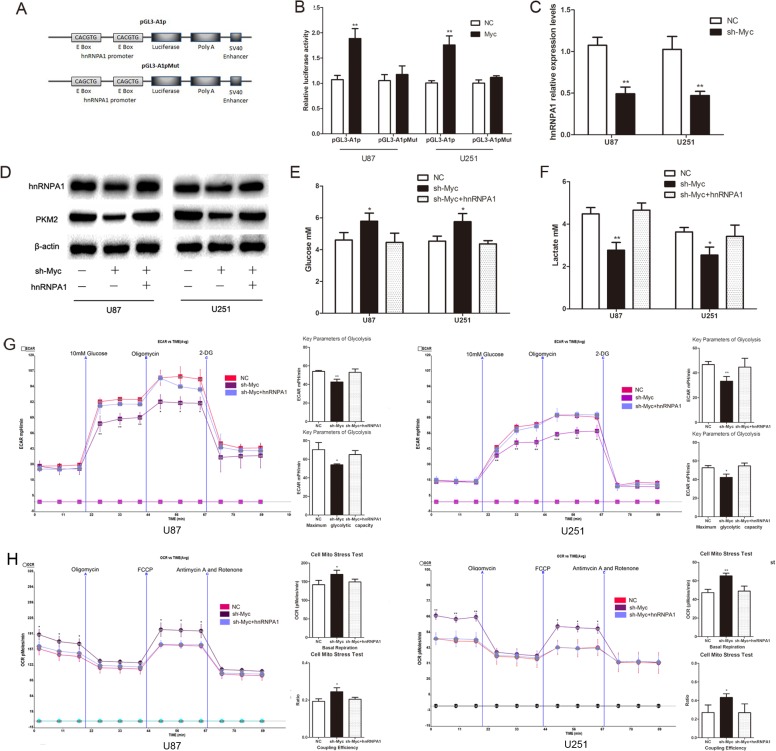
C-Myc regulates PKM2 via direct transcription of hnRNPA1 in glioma cells (A) The hnRNPA1 promoter-luciferase reporter construct, pGL3-A1p, contains E boxes (CACGTG), which are located within a ~700nt hnRNPA1 promoter region and serve as putative c-Myc binding sites. The mutant construct, pGL3-A1pMut, has a mutated sequence within the E Box (CAGCTG). [12] (B) Over-expression of c-MYC in U87 and U251 glioma cells led to a marked increase in the luciferase activity of pGL3-A1p, without any change in the luciferase activity of pGL3-A1pMu. NC, negative control vector. (C) The expression of HnRNPA1 was detected using qRT-PCR following transfection with sh-Myc. (D) Western blots identified hnRNPA1 and PKM2 expression changes following transfection with sh-Myc alone or in combination with hnRNPA1. β-actin is shown as a loading control. (E and F) The concentration of glucose and lactate in the culture medium was measured after the glioma cells were transfected with sh-Myc alone or in combination with hnRNPA1. (G and H) ECAR and OCR were measured by the Glycolysis Stress and Cell Mito Stress tests in glioma cell lines. *P < 0.05, **P < 0.01, ***P<0.001. Results are representative of at least three independent experiments.

The E boxes (CACGTG), which are located within a ~700nt hnRNPA1 promoter region, are putative c-Myc binding sites.[12] To confirm the direct interaction between c-Myc and hnRNPA1, we tested pGL3-A1p (wild-type E boxes) and pGL3-A1pMu (mutated E boxes) promoter-luciferase reporter constructs (Fig. 4A)[12]. Dual luciferase reporter assays showed that c-Myc overexpression led to a marked increase in the luciferase activity of pGL3-A1p, without any change in pGL3-A1pMu (Fig. 4B). QRT-PCR shows that the expression of hnRNPA1 is low in c-Myc reduction group (Fig. 4C). Furthermore, reduction of c-Myc expression (Supplementary Fig.2) inhibited hnRNPA1 and PKM2 expression, glucose consumption and lactate generation; though these effects each were reversed by concomitant hnRNPA1 expression (Fig. 4D-F). Consistently, cells with c-Myc repression showed lower ECAR and higher OCR levels, which also were reversed by hnRNPA1 expression (Fig. 4G and Fig. 4H). These results suggest that c-Myc drives the expression of hnRNPA1, which in turn promotes PKM2-mediated glycolytic activity.

### C-Myc is a functional target of let-7a that affects glucose metabolism in glioma cells

**Figure 5:**
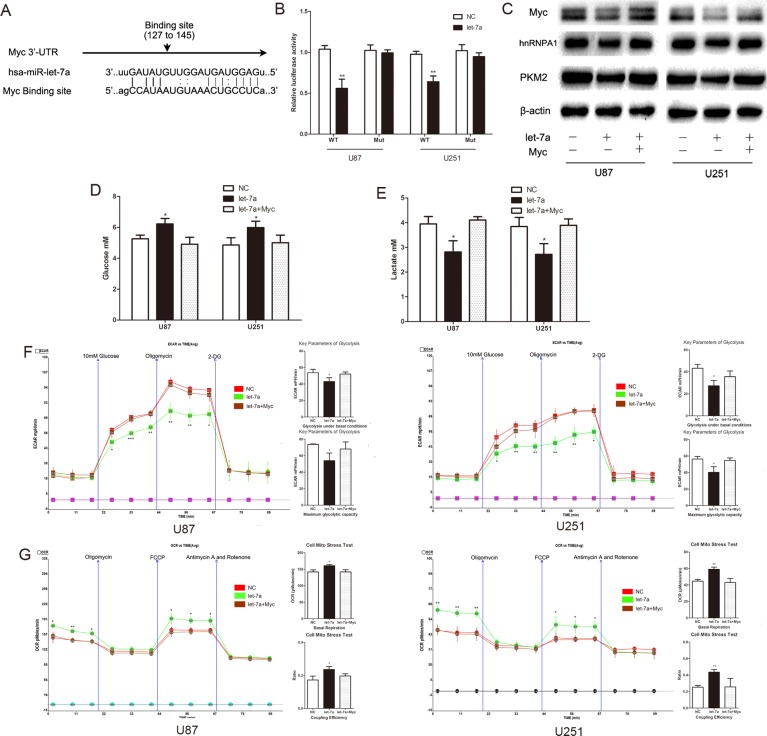
C-Myc is a functional target of let-7a that affects glucose metabolism in glioma cells (A) Putative binding sites of Myc within the 3′UTR, as predicted by miRanda. (B) Over-expression of let-7a in U87 and U251 glioma cells led to a marked decrease in the luciferase activity of pGL3-WT-c-Myc-3′UTR-Luc, without any change in the luciferase activity of pGL3-mut–c-Myc-3′UTR-Luc. NC, negative control miRNA. (C) Western blots identified Myc, hnRNPA1 and PKM2 expression a change following transfection with let-7a alone or in combination with Myc. β-actin is shown as a loading control. (D and E) The concentration of glucose and lactate in the culture medium was measured after the glioma cells were transfected with let-7a alone or in combination with Myc. (F and G) ECAR and OCR were measured in glioma cell lines after transfection with let-7a alone or in combination with Myc. *P < 0.05, **P < 0.01, ***P<0.001. Results are representative of at least three independent experiments.

Using miRNA target prediction we determined that c-Myc is a potential target of let-7a (Fig. 5A). To verify the ability of let-7a to target c-Myc at the predicted 3′-UTR site, we generated constructs containing wild-type (pGL3-WT-c-Myc-3′UTR-Luc) and mutant (pGL3-mut–c-Myc-3′UTR-Luc) binding sites of the c-Myc 3′UTR. Dual luciferase reporter assays showed that let-7a over-expression led to a marked decrease of luciferase activity of pGL3-WT-c-Myc-3′UTR-Luc, without any change to pGL3-mut–c-Myc-3′UTR-Luc (Fig. 5B). Western blotting showed that c-Myc was decreased in let-7a mimic-transfected cells, with downstream effects on hnRNPA1 and PKM2 expression; and that the reduction in the levels of these proteins was reversed by c-Myc overexpression (Fig. 5C). These data indicate that c-Myc is a functional target of let-7a. The effect of let-7a on glucose metabolism was also largely abrogated by c-Myc overexpression plasmid in co-transfected glioma cells (Fig. 5D-G), suggesting that the activity of let-7a in repressing the glucose metabolism of glioma cells may be mediated through its repression of c-Myc.

### HnRNPA1 can act as a repressor of let-7a biogenesis

**Figure 6:**
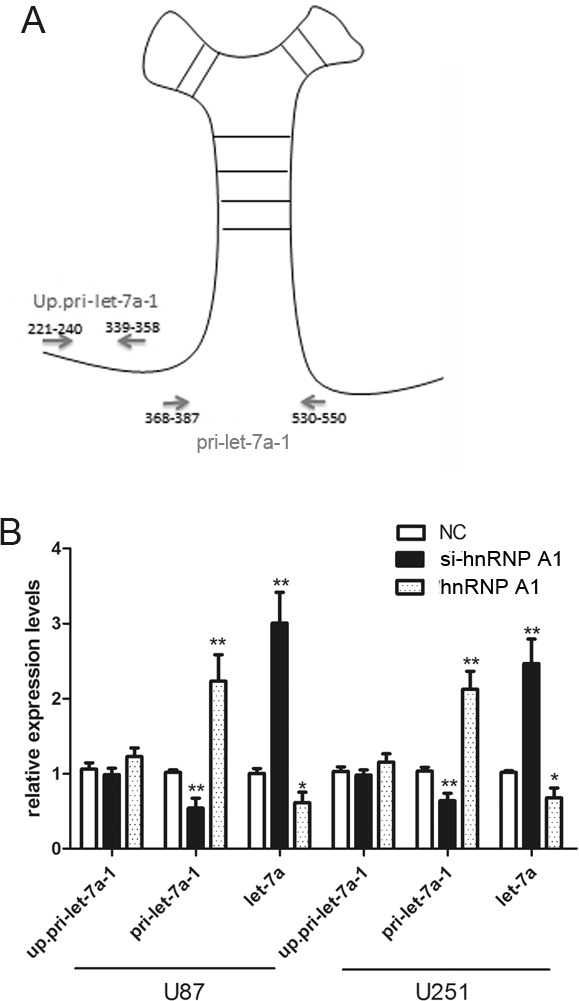
HnRNPA1 can act as a repressor of let-7a biogenesis in glioma cells (A) Specific primers were designed to cover either the upstream region (Up.pri-let-7a-1) as a measure the transcription of the pri-miRNA; or to span the pri-let-7a-1 stem loop structure (pri-let-7a-1) as a measure of the levels of unprocessed pri-miRNA. [14]. (B) The expression of up. pri-let-7a-1, pri-let-7a-1 and mature let-7a were determined by qRT-PCR after transfection of si-hnRNPA1 or hnRNPA1 or a negative control vector (NC) into U87 and U251 cells. Expression was normalized to the expression of U6 rRNA. *P < 0.05, **P < 0.01. Results are representative of at least three independent experiments.

Recognition and cleavage of primary microRNA precursors by the nuclear processing enzyme Drosha is a critical step during human microRNA biogenesis. HnRNPA1 binds to the conserved terminal loop of pri-let-7a-1 and blocks its processing by Drosha.[14] To determine whether hnRNPA1 can repress let-7a biogenesis in glioma cells, we designed specific primers to detect either the upstream region or the pri-let-7a-1 stem loop structure (Fig. 6A). The upstream primers serve as a measure of the change in transcription levels by probing both un-cleaved and cleaved pri-let-7a-1 transcripts, while the primers spanning the pri-let-7a-1 stem loop structure only detected the un-cleaved pri-let-7a-1 transcripts.[14] qRT-PCR with up.pri-let-7a-1 primers showed that modulation of hnRNPA1 levels by siRNA-mediated knockdown or hnRNPA1 overexpression did not significantly change the rate of let-7a transcription (Fig. 6B, up.pri-let-7a-1). However, modulation of hnRNPA1 resulted in a corresponding change in the levels of unprocessed pri-let-7a-1 (have not been cleaved by Drosha), with the reverse effects on the levels of mature let-7a (Fig. 6B, pri-let-7a-1 and let-7a). These date indicate that hnRNPA1 inhibits let-7a expression in glioma cells, and that the inhibition occurs at the level of biogenesis.

### Let-7a inhibits the expression of c-Myc, hnRNPA1 and PKM2 *in vivo*

**Figure 7:**
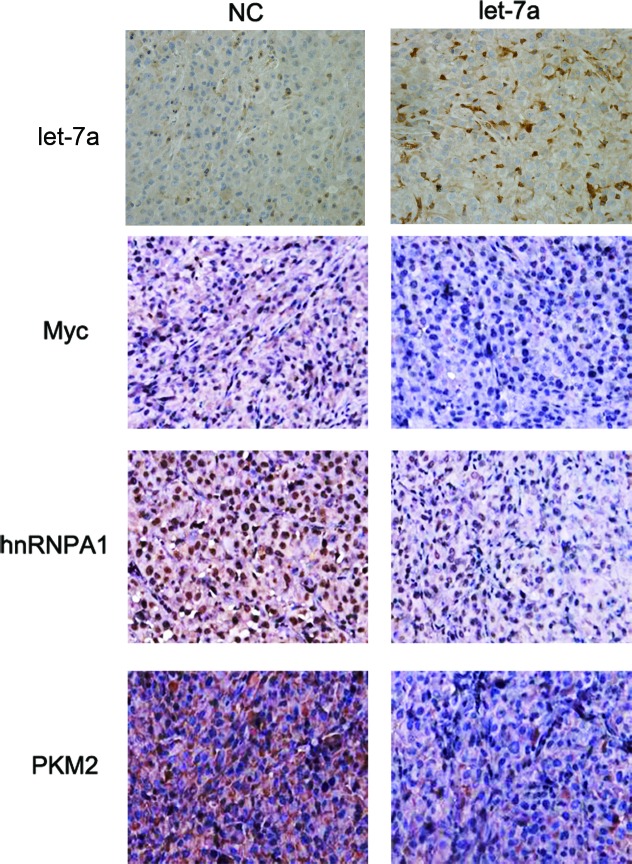
Let-7a inhibits glioma growth *in vivo* The expression of let-7a, c-Myc, hnRNPA1 and PKM2 was examined by Immunohistochemical and In situ hybridization staining of sections from a glioma xenograft model in nude mice. The proteins were stained with ABC-peroxidase and diaminobenzene (brown color), while the cells were counterstained with hematoxylin (blue). Results are representative of at least three independent experiments.

Our previous results have shown that let-7a inhibits glioma cells in a glioma xenograft model.[13] Thus, the expression of c-Myc, hnRNPA1 and PKM2 was examined in glioma tissues. The expression of these proteins was reduced in the let-7a mimic-treated group (Fig. 7). We also detected the expression of let-7a, c-Myc, hnRNPA1 and PKM2 in glioma tissues by qRT-PCR. The expression of let-7a was lower in glioma tissues than in normal brain tissues, and negative correlated with glioma grade. However, the expression of c-Myc, hnRNPA1 and PKM2 is opposite (Supplementary Fig.3). These results provide *in vivo* demonstration of the role of let-7a in the regulation of the c-Myc/hnRNPA1/PKM2 pathway in glioma.

## DISCUSSION

Recent studies have shown that pyruvate kinase, the enzyme that catalyzes the last step of glycolysis (phosphoenolpyruvate conversion to pyruvate),[7] functions as a regulator of cancer cell metabolism, which is exemplified high glucose consumption and lactate production.[15] There are four isoforms of pyruvate kinase in mammalian cells (PKR, PKL, PKM1, PKM2).[16] While PKM1 is expressed in skeletal muscle, heart and brain, the PKM2 isoform is expressed in embryonic development and is almost universally re-expressed in cancer cells.[9, 17] Unlike PKM1, PKM2 mainly promotes aerobic glycolysis. When PKM2 is replaced by PKM1 in cancer cells, the lactate production and proliferation ability are low, while oxygen consumption is increased.[9] The activity of PKM2 is low and leads to the accumulation of glycolytic intermediates that can be used for biosynthesis, which may be favorable for cell growth.[17-20] In addition, PKM2 also participate in glutamine metabolism though promoting the signaling pathway of β-catenin and enhancing its downstream c-Myc-mediated glutamine metabolism.[21] Recent data have shown that PKM2 binding with TGIF2 recruits histone deacetylase 3 to the E-cadherin promoter, thereby promoting epithelial–mesenchymal transition (EMT).[22]

Few studies fuscous on the role of PKM2 in glioma cell metabolism. In the current study, we show that PKM2 is critical for aerobic glycolysis and growth of glioma cells. PKM2 knockdown induced significant inhibition of glucose consumption, lactate generation. To further reveal the dynamic glycolysis ability of PKM2, Glycolysis Stress and Cell Mito Stress tests were employed. ECAR and OCR have been documented to be key indicators of glycolytic capacity and mitochondrial respiration, respectively. ECAR and OCR data showed that PKM2 repression inhibited the rate of glycolysis under basal conditions and maximum glycolytic capacity, and upreguated the basal respiration and the coupling efficiency.

PKM contains 12 exons, and the distinction between PKM1 and PKM2 is the existence of exon 9 in the M1 isoform or exon 10 in the M2 isoform. These two isoforms result from alternative splicing of the PKM transcript, and this splicing is controlled by the hnRNP family (hnRNPA1, hnRNPA2 and PTB), which repressively binds the sequences flanking exon 9 and promotes the formation of PKM2.[12, 23, 24] C-Myc up-regulates transcription of hnRNP family proteins and increases the PKM2/PKM1 ratio in many cancers.[12] Here, we demonstrate that hnRNPA1 contributes to the generation of PKM2 but inhibits PKM1 production in glioma cells. The effects of c-Myc in increasing PKM2 expression are mediated through its up-regulation of hnRNPA1 in glioma cells. Previous studies have suggested several other factors that can modulate PKM2 expression. Sun et al demonstrated that the PI3K/AKT/mTOR pathway up-regulates PKM2 through both hypoxia-inducible factor 1α (HIF1α)-mediated transcription activation and c-Myc/hnRNPs-dependent regulation of PKM2 gene splicing.[8]

The function and mechanism of microRNAs in tumorigenesis has been well studied. Our previous data demonstrate that let-7a is a tumor suppressor in glioma.[13] In this study, our data indicate that c-Myc is a functional target of let-7a that affects glucose metabolism in glioma cells. The processing of the primary microRNA transcript by the nuclear processing enzyme Drosha results in the generation of the precursor microRNA hairpin (pre-miRNA).[25, 26] Then the pre-miRNA is exported to the cytoplasm and is processed by the type III rib nuclease Dicer, resulting in the generation of the mature miRNA.[27] The splicing factor KH-type splicing regulatory protein (KSRP) is a component of both Drosha and Dicer complexes that promotes the biogenesis of a subset of miRNAs, including let-7.[28, 29] hnRNPA1 binds to the terminal loop of pri-let-7a-1, blocking its processing by Drosha and decreasing let-7a production. Thus hnRNPA1 and KSRP play antagonistic roles in the biogenesis of let-7a.[14] In this study, we demonstrate that hnRNPA1 acts as a repressor of let-7a biogenesis in glioma cells. Our results suggest that let-7a, c-Myc and hnRNPA1 form a feedback loop to modulate PKM2 expression, therefore regulating glioma cell glucose metabolism (Supplementary Fig.4).

In conclusion, we demonstrated that PKM2 is a key regulator of glucose metabolism in glioma. Our study provides the first report to demonstrate that the let-7a/c-Myc/hnRNPA1 feedback loop contributes to PKM2 expression and glucose metabolism. Understanding the regulation of this signaling pathway in glioma could lead to the identification of new therapeutic targets for treating glioma. Future studies to assess the role of the let-7a/c-Myc/hnRNPA1/PKM2 pathway in a clinical context are warranted.

## METHODS

### Tissue samples

All human glioma samples and normal brain tissue were obtained from the patients who diagnosed with WHO(World Health Organization,2007) grade II-IV glioma. They underwent surgical resection at Department of Neurosurgery of the First Affiliated Hospital of Nanjing Medical University. After collection, every glioma tissue was immediately frozen in liquid nitrogen. We collect 25 specimens in liquid nitrogen, including 5 normal brain tissues, 10 low grade glioma, and 10 high grade glioma.

### Cell lines and cell culture

The human U87, U251, H4 and T98G glioma cell lines were purchased from the Chinese Academy of Sciences Cell Bank (Shanghai, China). The cells was cultured in Dulbecco's modified Eagle's medium (DMEM) (Gibco, Los Angeles, USA), supplemented with 10% fetal bovine serum (Invitrogen), and were incubated in an atmosphere containing 5% CO2 at 37 °C.

### Oligonucleotides, plasmids and transfection

Oligonucleotides were chemically synthesized by GenePharma (Shanghai, China). The sequences are as follows: hsa-miR-let-7a mimic, 5′-UGAGGUAGUAGGUUGUAUAGUU-3′; hnRNPA1- small interfering RNA (siRNA) (si-hnRNPA1), 5′-CAGCUGAGGAAGCUCUUCATT-3′; PKM2-siRNA (si-PKM2), 5′-CCAUAAUCGUCCUCACCAATT-3′; negative control (NC), 5′-UUCUCCGAACGUGUCACGUTT-3′. The c-Myc–short hairpin RNA (shRNA) (shMyc) oligonucleotide was designed and cloned into vector U6/GPF/Neo by GenePharma, based on the following sequences: human c-Myc-shRNA, 5′-CACCGCCATAATGTAAACTGCCTCAACTC GAGTTGAGGCAGTTTACATTATGGTTTTTG-3′; negative control (NC), 5′-CACCGTTCTCCGAACGTGTCACGTCAAGAGA TTACGTGACACGTTCGGAGAATTTTTTG-3′. The pET9d-hnRNP-A1 and pMSCV-Flag-cMyc T58A plasmids were obtained from Addgene (USA). Oligonucleotides and plasmids were transfected into glioma cells using Lipofectamine 2000 (Invitrogen). Oligonucleotides or plasmids were complexed with Lipofectamine 2000 by OPTI-MEM (Invitrogen) and added to cells. The medium was changed after 6-8 h incubation.

### CCK-8 proliferation assay

The proliferative ability of glioma cells was assessed using CCK-8 (Beyotime, China). The U87 and U251 cells were seeded into 96-well cell culture plates. Every well contained 5×10^3^ cells in 100 μl culture media. Subsequently, hsa-miR-let-7a mimic, si-PKM2 or negative control siRNA were transfected into cells. After 24 h, 48 h, 72 h or 96 h, the medium of each well was replaced with 100 μl fresh medium with 10 % CCK8, and then the cells were incubated at 37°C for an additional 3 h. The absorbance was measured at 450 nm wavelength.

### The concentration of glucose and lactate measurement

Glucose and Lactate Assay Kit (Biovision, USA) were used to measure the glucose and lactate concentrations of the culture medium. After the cells were transfected for 48 h, 2 μl of medium was added into a series of well on a 96-well plate. The standard solution was diluted to 1 nmol/μl using assay buffer. Subsequently, 0, 2, 4, 6, 8 or 10 μl of standard solution were added into the other blank well, and the volume was adjusted to 50 μl/well with assay buffer to generate 0, 2, 4, 6, 8 or 10 nmol/well final concentration of glucose or lactate standard. Finally, 50 μl of the reaction mix were added to each well and the plates were incubated for 30 minutes at 37°C. The optical density was measured at 570 nm wavelength, and the sample readings were applied to the standard curve to calculate the glucose and lactate concentrations of the test samples.

### Glycolysis stress test

The extracellular acidification rate (ECAR) was measured using the Seahorse XF96 Analyzer Glycolysis which calculates the net production and extrusion of protons into the extracellular medium. As glycolysis occurs, the resulting acidification of the medium surrounding the cells is measured directly by the XF Analyzer and reported as the ECAR. Initially, cells are incubated in glycolysis stress test medium without glucose. The ECAR refers to non-glycolytic acidification, which includes CO_2_ evolution followed by its hydration to carbonic acid and bicarbonate, as well as proton extrusion. The first injection is a saturating concentration of glucose. Glucose is taken up by the cells and catabolized to lactate, producing ATP and protons, with a corresponding rapid increase in ECAR. This glucose-induced response is reported as the rate of glycolysis under basal conditions. The second injection is oligomycin. It inhibits mitochondrial ATP production and thus shifts the energy production to glycolysis, with the increase in ECAR revealing the maximum glycolytic capacity. The final injection is 2-DG, a glucose analog, which inhibits glycolysis through competitive binding to glucose hexokinase. The resulting decrease in ECAR further confirms that the ECAR produced in the experiment is due to glycolysis. The difference between the Glycolytic Capacity and Glycolysis Rate defines the Glycolytic Reserve.

### Cell mito stress test

The oxygen consumption rate (OCR), an indicator of mitochondrial respiration, was measured by using the Seahorse XF96 Analyzer (Seahorse Bioscience, USA). The cells are metabolically perturbed by the addition of three different compounds in succession, which shifts the bioenergetics profile of the cell. The basal OCR was measured and predominantly controlled by the parallel re-entry pathways through ATP synthase and leakage. The first injection was oligomycin, an ATP synthase inhibitor. The decrease of OCR on adding Oligomycin approximates the proton current flowing through ATP synthase. The decrease relative to the basal level provides the coupling efficiency. The residual respiration is due to proton leakage. The second injection is FCCP, an ionosphere that is a mobile ion carrier and introduces a high artificial proton conductance into the membrane, which leads the a rapid consumption of oxygen. This maximal respiration is controlled by electron transport chain activity and/or substrate delivery. The increased respiratory capacity above basal respiration provides spare respiratory capacity. Finally, electron transport chain inhibitors (Rotenone and Antimycin A) are added. Any residual respiration is non-mitochondrial and needs to be subtracted from the other rates.

### RNA extraction and quantitative RT –PCR

RNA was extracted from cells and tissues using TRIzol (Invitrogen, USA), according to the manufacturer's protocol. To detect the cellular levels of let-7a, exon 9, exon 10, pri-let-7a-1 and the upstream region (up.) of pri-let-7a-1, reverse transcription (RT) was conducted with the Applied Biosystems® TaqMan® MicroRNA Reverse Transcription Kit (Applied Biosystems, CA) and Fermentas reverse transcription reagents. The primers for the let-7a were synthesized and purchased from Guangzhou RiboBio BioCoLTD (Guangzhou, China). U6 was used for normalization. To assess the levels of c-Myc, PKM2, hnRNPA1, exon 9, exon 10, pri-let-7a-1 and up.pri-let-7a-1, GAPDH was used for normalization. Primers as follows: pri-let-7a-1 forward CCTTCCTGTGGTGCTCAACT, pri-let-7a-1 reverse CTTTCTATCAGACCGCCTGGA; up.pri-let-7a-1 forward CAGGAAATGAAACCACAGCA, up.pri-let-7a-1 reverse CCTCCTCGGTAATCCTGGTT; exon 9 forward CCCCTCTTCCCCTAAACCTT, exon 9 reverse TGGAGCAAGAGGCTGGTTAT; exon 10 forward CTTCTGTATGTCCCCCATCC, exon 10 reverse TCTAGGCTCTAGCCCCTGCT ; c-Myc forward TTCTCTCCGTCCTCGGATTC, c-Myc reverse GTAGTTGTGCTGATGTGTGG; hnRNPA1 forward CAGATAAAGGCCCTCTTTCCC, hnRNPA1 reverse CTCAGCTACATTAGGGTTATTGGG; PKM2 forward CTGTGGACTTGCCTGCTGTG, PKM2 reverse TGCCTTGCGGATGAATGACG. ABI Step One Plus was used to perform the ampliﬁcation reaction. Result was analyzed using the 2^−ΔΔCt^ method. Each sample was run in triplicate.

### Western blot analysis

Proteins were extracted from cells after transfection using RIPA lysis buffer (KenGEN, China). Protein concentrations were determined with a BCA Protein Assay Kit (Beyotime, China). Equal amounts of protein per lane were separated by 10% SDS-PAGE and transferred to PVDF membranes (Millipore, USA). Subsequently, membranes were blocked in 5% skim milk for 1 h and incubated overnight at 4°C with diluted antibodies against c-Myc (1:10000, Abcam, UK), hnRNPA1 (1:1000, Abcam, UK), PKM2 (1:1000, Cell Signaling Technology, USA), or PKM1 (1:1000, Sigma, USA). Finally, the membranes were incubated with HRP-conjugated secondary antibody (1:2500, Santa Cruz, USA). β-actin was used as a control (Santa Cruz, USA).

### Luciferase reporter assay

The 3′-UTR fragment of c-Myc containing the let-7a binding sequences and the fragment of the hnRNPA1 promoter containing c-Myc putative binding sites were cloned into firefly luciferase reporter construct. Mutated plasmid was used as control. PGL3-WT-c-Myc-3′UTR-Luc, pGL3-mut–c-Myc-3′UTR-Luc, pGL3-A1p and pGL3-A1pMu were constructed by Invitrogen (USA). The cells were co-transfected with luciferase reporter construct, hsa-miR-let-7a mimic or c-Myc expression vector. Cells were collected after 24 h transfection and luciferase activity was measured using the Dual Luciferase Reporter Assay System (Promega, USA).

### Xenograft tumor assay

Twelve immunodeficient female nude mice were used to test whether let-7a could downregulate the c-Myc/hnRNPA1/PKM2 signaling pathway *in vivo*. All nude mice were kept at a constant humidity and temperature and bred in aseptic conditions according to standard guidelines under a protocol approved by Nanjing Medical University. The nude mice tumor xenograft model was performed as previously described. The nude mice were divided into the let-7a group and the scramble group. The mixture of 200 pmol let-7a mimic oligonucleotides and 10 μL Lipofectamine 2000 was locally injected into every xenograft tumor at multiple sites in let-7a group, in the scramble group was injected with 200 pmol of scrambled oligonucleotides and 10 μL Lipofectamine 2000. These injections were performed every 3 days. The tumors were measured and harvested as previously described and preparation of paraffin-embedded sections.[13]

### Immunohistochemistry staining and In situ hybridization

Xenograft tumor sections were incubated with primary antibodies against c-Myc (1:50, Abcam, UK), hnRNPA1 (1:100, Abcam, UK), or PKM2 (1:400, Cell Signaling Technology, USA) overnight at 4°C. Subsequently, the sections were incubated with a biotinylated secondary antibody (1:200, Gene Tech, China) at room temperature for 1 h, followed by incubation with ABC-peroxidase for 1 h, staining with with diaminobenzidine for 5 min and counterstaining with hematoxylin (Gene Tech, China). The results were assessed by two experienced pathologists in a blind manner. In every section, five randomly selected visual fields were assessed by 200 light microscopes to evaluate the effect of increasing let-7a expression on the expression of c-Myc, hnRNPA1 and PKM2. Using antisense locked nucleic acid (LNA)-modified probes (Boster, Wuhan, China), *in situ* hybridization was performed. Oligonucleotide sequences were: LNA- let-7a, 5′-ACTCCATCATCCAACATATCTT-3′. Sections with no labeling or with fewer than 5% labeled cells were scored as 0. Sections with 5%-30% of cells labeled were scored as 1, with 31%-70% of cells labeled as 2, and with labeling of ≥71% as 3. The staining intensity was scored similarly, with 0 used for negative staining, 1 for weakly positive, 2 for moderately positive, and 3 for strongly positive. The scores for the percentage of positive tumor cells and for the staining intensity were added to generate an immunoreactive score for each specimen. The product of the quantity and intensity scores were calculated such that a final score of 0-1 indicated negative expression (−), 2-3 indicated weak expression (+), 4-5 indicated moderate expression (++), and 6 indicated strong expression (+++). Each sample was examined separately and scored by 2 pathologists. Cases with discrepancies in the scores were discussed to reach a consensus.[30]

### Statistical analysis

All experiments were performed three times and data were presented as mean ± standard error. Data were analyzed with SPSS 10.0. T-test was used to analyze differences in each two-group comparison, while One-way ANOVA was used to determine the difference among at least three groups. *P* < 0.05 was considered statistically significant.

## SUPPLEMENTARY MATERIALS FIGURES


