# Supplementary material for: PKM2 promotes glucose metabolism and cell growth in gliomas through a mechanism involving a let-7a/c-Myc/hnRNPA1 feedback loop
Source: Oncotarget. 2015 Feb 10;6(15):13006–18. doi: 10.18632/oncotarget.3514 (PMC4536995; doi:10.18632/oncotarget.3514)
Supplement: Supplementary file 1 [file oncotarget-06-13006-s001.pdf]

PKM2 promotes glucose metabolism and cell growth in gliomas through a mechanism involving a let-7a/c-Myc/hnRNPA1 feedback loop

Supplementary Material

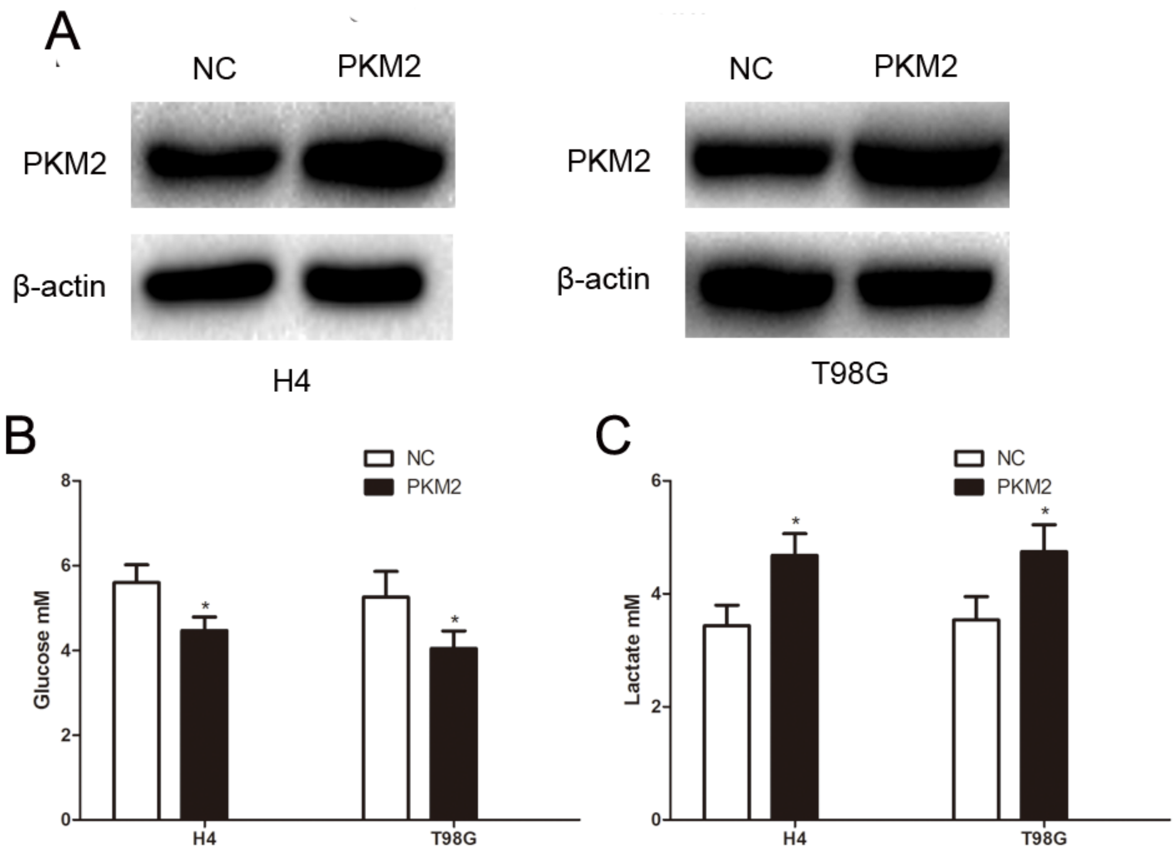

Supplementary Fig.1

(A) Transfection efficiency of PKM2 plasmid was determined by Western blotting in H4 and T98G.

(B and C) The concentration of glucose and lactate in the culture medium was measured.

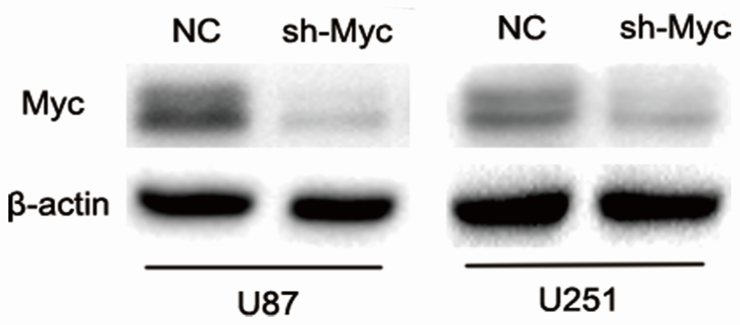

Supplementary Fig.2

Transfection efficiency of sh-Myc was determined by Western blotting. β-actin is shown as a loading control. Results are representative of at least three independent experiments.

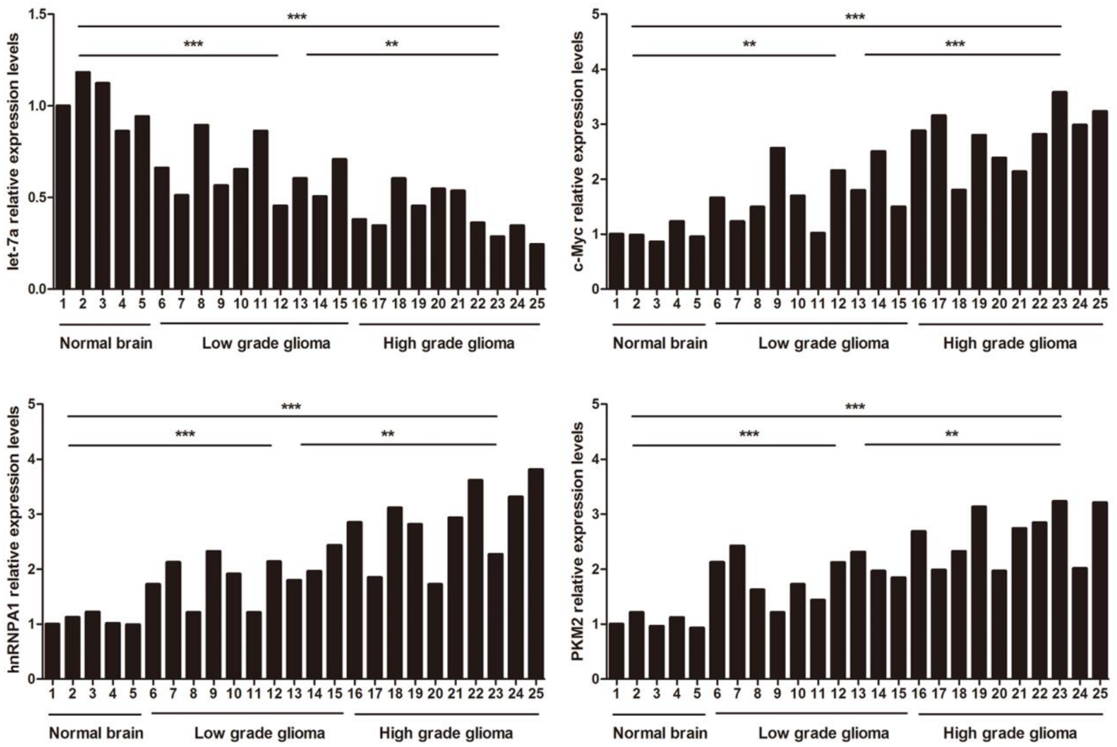

Supplementary Fig.3

The expression of let-7a, c-Myc, HnRNPA1 and PKM2 in glioma tissues and normal brain tissues was detected by qRT-PCR.

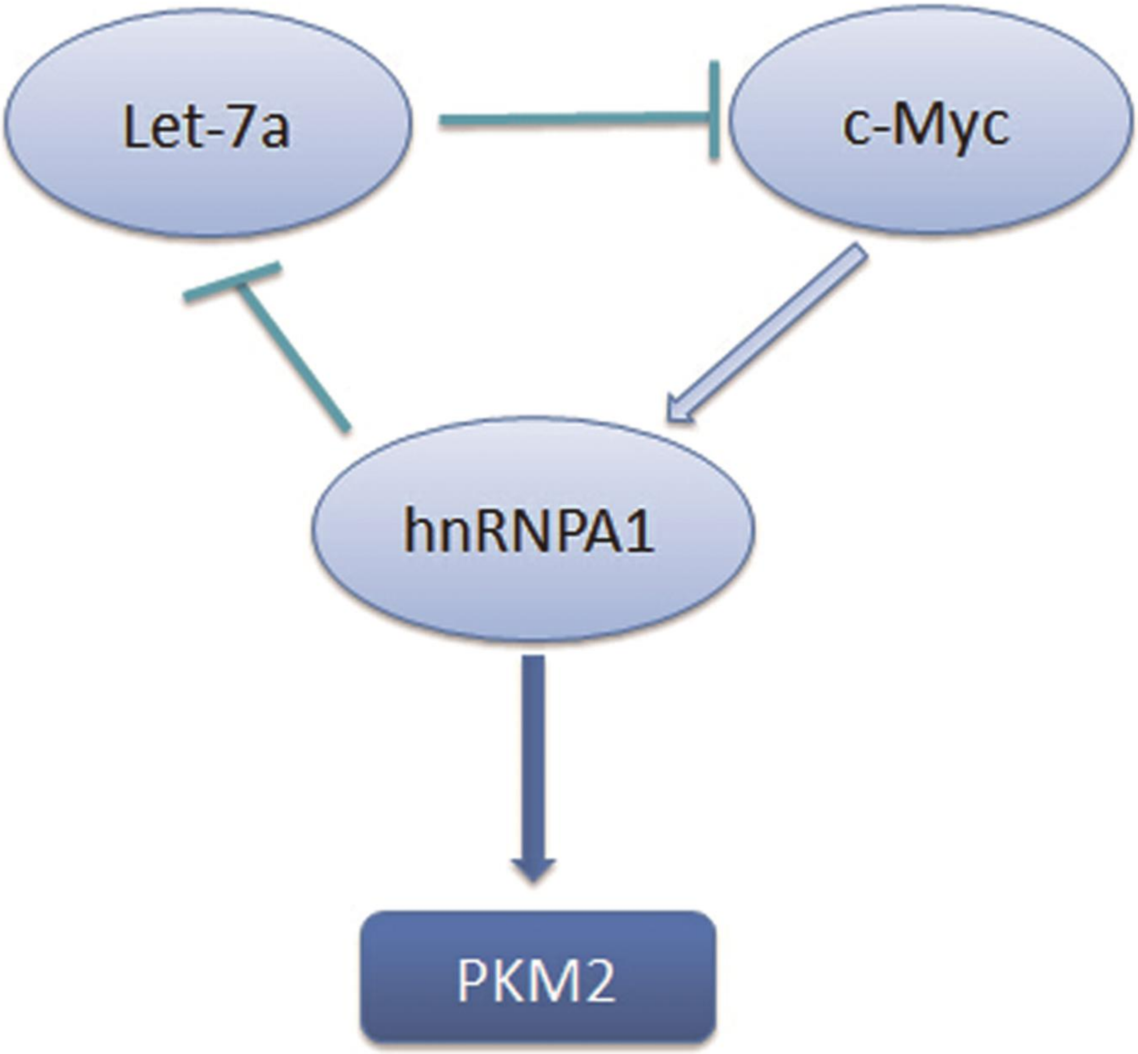

Supplementary Fig.4

The schematic diagram of the feedback loop
